# Development and Validation of the Healthcare Expenditure and Location Dynamics in Acute Febrile Illness (HEAL-AFI) Tool: A Community-Based Cohort Study With a Special Focus on Dengue and Chikungunya in the Urban Slum of Delhi, India

**DOI:** 10.7759/cureus.105972

**Published:** 2026-03-27

**Authors:** Nandini Sharma, Shivani Rao, Madhvi Dhamania, Nidhi Bhatnagar, Himanshu Bachawandia, Mongjam M Singh

**Affiliations:** 1 Community Medicine, Maulana Azad Medical College, New Delhi, IND

**Keywords:** cost of illness, dengue, direct cost, economic burden, fever, health expenditure, indirect cost, reliability, urban slums, validity

## Abstract

Introduction: Cost-of-illness (COI) studies quantify the economic burden of diseases, yet existing tools often lack standardized cost domains and contextual adaptation. We developed and validated the Cost of Healthcare Expenditure Questionnaire (CHEQ), a generic tool, and its adaptation for acute febrile illness (HEAL-AFI), tailored to dengue and chikungunya in Delhi slum communities.

Methodology: Conducted in a resettlement colony of Northeast Delhi, this study followed a multistage process with five iterative rounds. Round 1 involved item generation after the literature review. In Round 2, eight experts assessed content validity using Lawshe’s method. Round 3 reassessed content validity and translated the tool. Round 4 tested face validity and feasibility among 32 participants and eight interviewers. Round 5 evaluated reliability via test-retest after 12 days using Cohen’s κ, intraclass correlation coefficients (ICCs), and Cronbach’s α. Data were collected on KoboCollect and analyzed in IBM SPSS Statistics for Windows, version 26.0.

Results: Expert review retained 74 items for CHEQ (76 for HEAL-AFI), with CVI improving from 0.78 (Round 2) to 0.92 (Round 3). Face validity demonstrated good clarity and ease of use, with a mean administration time of 24.3 ± 5.1 minutes (T1) and 23.7 ± 4.8 minutes (T2). Reliability was substantial for categorical variables (κ = 0.63-0.79; agreement 72-84%) and excellent for continuous variables (ICC = 0.82-0.94). Internal consistency was high across domains (Cronbach’s α = 0.78-0.89).

Conclusion: CHEQ and HEAL-AFI are validated, reliable, and feasible tools for capturing household healthcare expenditures. CHEQ provides a standardized framework across diseases, while HEAL-AFI contextualizes costs for acute febrile illnesses (AFIs) with geospatial integration. These tools can strengthen COI research and may guide equitable health policy.

## Introduction

Cost-of-illness (COI) studies are designed to comprehensively capture the economic costs associated with a disease [1]. By quantifying the financial burden of illness on society, they provide estimates of the potential savings from prevention or elimination, which can inform policymakers on priority setting, planning, and resource allocation [2]. Therefore, evidence on the magnitude and distribution of illness-related costs is essential for developing efficient and equitable health policies, especially under constrained budgets [3].

COI studies convert the clinical and social consequences of disease into monetary values, thus quantifying direct expenditure and productivity losses at the household and societal level [4]. However, most COI research focuses on direct medical and non-medical expenses, with intangible costs rarely included due to measurement difficulties [5]. Although the strength of COI studies lies in presenting intuitive, forthright policy-relevant data, the methodological challenges persist, particularly around defining cost domains, minimizing recall bias, and ensuring contextual adaptation for diverse diseases and settings. Many Indian and international studies are narrow in scope, hospital-based, age-specific, or limited to single disease entities such as typhoid, diabetes, or rheumatoid arthritis [6-11]. In addition, inconsistent use of cost categories is common; for instance, direct non-medical costs are sometimes grouped with indirect costs, limiting comparability [6,8,9]. Even the evidence regarding the economic burden of dengue and chikungunya in India remains fragmented, incomplete, and highly variable in methodology. A systematic review noted wide heterogeneity in dengue costing approaches, definitions of expenditure categories, and recall periods, making cross-study comparisons difficult and underlining the need for standardized tools, especially for acute febrile illnesses (AFIs) such as dengue [12,13]. The consequences of AFI are particularly severe for socio-economically disadvantaged populations living in urban slums, who frequently rely on providers with limited or no formal medical training [14]. A validated, adaptable tool for capturing direct, indirect, and non-medical costs in community settings is therefore urgently required.

However, structured instruments to capture patient and household costs remain scarce. Tools developed for disease-specific applications may miss cost items that could apply to other conditions [15]. The Comprehensive Patient Costs Questionnaire (CoPaQ) was one of the first generic tools to address this gap [15]. However, its limitations included a lack of standardized domains of costs (direct medical, direct non-medical, indirect costs), a recall period that risked omitting small but frequent expenses, uncertain feasibility, and limited validation. Moreover, guidance on final cost calculation was also absent, overall restricting its adaptability and reliability across diverse health conditions and settings [15].

To address these gaps, we developed the Cost of Healthcare Expenditure Questionnaire (CHEQ), a generic instrument designed to systematically capture three key expenditure domains: direct medical, direct non-medical, and indirect costs. CHEQ provided a standardized and flexible framework applicable across illnesses. Building on this foundation, we further created the Healthcare Expenditure and Location Dynamics in Acute Febrile Illness (HEAL-AFI) questionnaire, an adaptation tailored to the epidemiology of dengue and chikungunya in urban slum communities of Delhi. HEAL-AFI retained the structured expenditure domains of CHEQ but introduced additional modules, sequelae, and integrated geospatial mapping to link expenditure patterns with healthcare utilization and socio-economic status. This integration enabled visualization of illness clusters, healthcare-seeking pathways, and inequities in access, an especially relevant approach for communicable, environmentally linked, and socio-economically stratified diseases such as dengue and chikungunya.

The aim of this study was to design, develop, and validate two instruments: the generic CHEQ and its disease-specific adaptation, the HEAL-AFI. CHEQ was intended to provide a standardized framework for capturing direct medical, direct non-medical, and indirect costs across illnesses, while HEAL-AFI contextualized these domains for AFI, particularly dengue and chikungunya, in urban slum settings of Delhi. The validation process focused on establishing content and face validity, assessing reliability and feasibility, and demonstrating the added value of integrating healthcare expenditure data with location dynamics to better understand patterns of healthcare-seeking and economic vulnerability.

## Materials and methods

Study setting

The study was conducted in an urban resettlement colony and adjoining slum areas of the Northeast District of Delhi, India. The site was purposively chosen because of its socioeconomic vulnerabilities, including low income, high population density, and inadequate housing conditions. It also functions as an established Demographic, Developmental, and Environmental Surveillance Site (DDESS), with prior household mapping, geocoding, and demographic profiling [16]. The population of approximately 55,000 individuals resides across Gokalpuri resettlement colony, Sanjay colony, Ganga Vihar, and Gokalpuri village. The area serves as the field practice site for a government medical college, enabling sustained community engagement and rapid survey implementation.

Study design

This study reports the development and cross-sectional expert validation of a modular instrument - the HEAL-AFI questionnaire - alongside the generic CHEQ. The instrument was developed within the context of a larger prospective, community-based cost-of-illness (COI) study nested in a multicentric cohort investigating the incidence and seroprevalence of AFI across 10 sites in India. The economic costing component was undertaken exclusively at the Delhi site. The modular instrument - the HEAL-AFI questionnaire - was designed, developed, and validated alongside the generic CHEQ. While CHEQ was conceptualized as a disease-agnostic tool for estimating patient and household costs using standardized domains for direct medical, direct non-medical, and indirect costs, HEAL-AFI was adapted specifically for acute febrile illnesses (AFIs), particularly dengue and chikungunya, in low-income urban settings. HEAL-AFI retained CHEQ’s core structure but introduced additional modules to capture dengue and chikungunya sequelae-related costs and to collect the geolocation data of households and their nearest healthcare facilities to enable GIS-based analyses. Dengue and chikungunya were selected as the focus because this validation study is part of a larger government-funded project targeting these illnesses, which are also among the most prevalent AFIs in the study setting.

Development of the CHEQ and HEAL-AFI questionnaires

Potential panelists were first approached via email with study information, and those who consented formed the expert panel. The questionnaire development followed a multistage process with five iterative rounds. The final questionnaire is given in the appendix.

Round-1: Item Generation

A draft questionnaire was prepared based on findings from prior COI tools [15,17-19]. The draft CHEQ and HEAL-AFI instruments comprised a total of six and seven sections, respectively. Section 1 captured sociodemographic characteristics; Section 2 included treatment-seeking behavior; Section 3 and Section 4 covered outpatient and inpatient costs, respectively, subdivided into direct medical and direct non-medical categories; Section 5 focused on indirect costs using the human capital approach; Section 6 captured average household expenditure and financing patterns; and Section 7 assessed sequelae-related costs for dengue and chikungunya.

Round 2: Content Validity

An expert panel of eight members, including community medicine specialists, epidemiologists, hospital administrators, and health economists, evaluated the draft instrument. Each item was rated for essentiality and relevance using Lawshe’s method [20]. The Content Validity Ratio (CVR) was calculated, with a minimum threshold of ≥0.75 applied, based on the panel size [21]. The Content Validity Index (CVI) was also computed for overall adequacy by averaging CVRs of retained questions with values above 0.80 considered acceptable [21]. In addition to these quantitative methods, panelists also provided qualitative feedback on cultural appropriateness, clarity of wording, and comprehensiveness of domains. Items falling below the threshold were revised or eliminated after consensus.

Round 3: Re-assessment of Content Validity and Translation

Revised drafts were re-circulated to the same panel. Items added or modified were re-evaluated for CVR and CVI. This ensured that retained questions met both quantitative thresholds and qualitative expectations. The final instrument was translated into Hindi and back-translated into English by independent bilingual experts. Discrepancies were resolved to ensure semantic equivalence. Local colloquial expressions (e.g., Bengali doctor, *jhaad phoonk* for traditional practitioner/spiritual healers) were incorporated to improve comprehension.

Round 4: Face Validity and Feasibility

Face validity and feasibility were tested among 32 purposively selected community participants with recent febrile illness, drawn from the same geographic area but outside the main cohort to avoid contamination while ensuring contextual similarity. Respondents assessed clarity and ease of understanding using a four-point Likert scale, while open-ended feedback captured difficulty in recall and item comprehension. Field investigators recorded administration time, respondent acceptability, and data completeness, and rated ease of administration also on a four-point Likert scale from “very easy” to “very difficult.” Feedback from participants and interviewers informed language simplification, use of local terms, insertion of filter questions to reduce timing, and refinement of item sequencing to match natural care-seeking sequences.

Round 5: Reliability

The same 32 participants were re-interviewed after an interval of 12 days to assess test-retest reliability [22]. Categorical variables (e.g., first point of care, facility type, financing source) were evaluated using Cohen’s kappa; continuous measures (e.g., cost amounts, travel time, and distance) were assessed using intraclass correlation coefficients (ICCs) [23,24]. Internal consistency of multi-item domains was estimated using Cronbach’s alpha, with α ≥ 0.70 considered acceptable. This combination of Cohen’s kappa, ICC, and Cronbach’s alpha ensured reliability testing across categorical, continuous, and multi-item domains, respectively.

Data collection and management

Eight trained field investigators conducted face-to-face interviews. They underwent two days of training prior to data collection for KoboCollect, including interviewing techniques and cultural sensitivity. The second day was focused on trial data collection and reviewing the feasibility, problems in schedule administration, and data entry. Real-time data entry was done electronically using KoboCollect, with embedded skip logic and range checks to minimize errors and missing data. Inpatient expenditures were cross-verified against hospital records or bills when available.

Data analysis

Responses from each round were synthesized in MS Excel (Microsoft® Corp., Redmond, WA), from where data were exported into IBM SPSS Statistics for Windows, version 26.0 (released 2019, IBM Corp., Armonk, NY) for cleaning and analysis. Descriptive statistics summarized participant sociodemographic characteristics and item-level response completeness. CVR, CVI, Cohen’s kappa, ICC, and Cronbach’s alpha were calculated with 95% confidence intervals. GIS outputs (household locations, facility types, and episode frequency) were generated using ArcMap 10.8.1 (Esri, USA).

Ethical considerations

The protocol received approval from the Institutional Ethics Committee of Maulana Azad Medical College and Associated Hospitals, New Delhi (F.1/IEC/MAMC/81/09/2020/No 289). Potential participants were approached at home, study objectives were explained in the local language, and written informed consent was obtained from adults. For children and adolescents, expenditure data were provided by a parent/guardian; assent was not sought for this validation exercise because financial data were collected from adults/guardians only. Confidentiality was maintained, and participation was voluntary.

## Results

Round 1: item generation and expert panel

Eight panelists participated in the expert review process, including five community medicine specialists (two with additional expertise in hospital administration and one with training in health economics), one economist, one public health researcher, and one medicine specialist. All were postgraduates, with ages ranging from 30 to over 60 years. Their diverse backgrounds ensured both technical rigor and contextual appropriateness in item generation. Through literature review and expert consultation, a total of 87 items were developed in Round 1.

Round 2: content validity

In Round 2, all 87 items were assessed using Lawshe’s content validity method [20]. With eight panelists, the minimum acceptable CVR was set at 0.75. A total of 64 items (73.6%) met this threshold. Fourteen items (16.1%) with low CVR or overlapping content were removed, and nine items (10.3%) were reworded or merged for clarity, resulting in a 73-item draft. Qualitative feedback from panelists, summarized in Table 1, guided revisions including simplification of medical terminology, adherence to standard cost definitions, and improved clarity and contextual alignment of cost-related items. Section 1 (sociodemographic) was excluded from the CVR/CVI analysis, as it contains factual items not subject to expert judgment. Section-wise CVR values in Round 2 ranged from 0.60 to 0.92, and the overall CVI was 0.78 (Table 2).

**Table 1:** Qualitative feedback from expert panelists

Section	Panelist feedback	Modifications
General	Simplify medical terminology; follow natural patient care-seeking pathway to aid recall	Medical terms simplified and question order aligned with typical care-seeking trajectory
Section 2: Treatment seeking behaviour	Add skip/filter questions; reduce overall administration time	Questions reworded and sequencing optimised to reduce time burden
Section 3: Subsection - Direct medical cost	Remove overlapping items on OPD medicines and investigations	Investigations added as separate item to avoid duplication
Section 3: Subsection - Direct non-medical cost	Broaden terminology (e.g., “traditional practitioner/spiritual healer”) to include local terms; include home remedies	Local terms such as Bengali doctor and jhaad phook added in brackets for clarity; home remedies included under “Special diet”; outside food consumed by caregivers during transit or care of patient included under “Miscellaneous”
Section 3 and Section 4	Add consolidated cost option to capture instances where participants could not provide detailed break-ups.	Consolidated cost option included
Section 5: Caregiver relation and employment status	Clarify caregiver relationship and their work/school status; children may also take caregiving roles	Relationship to care recipient and caregiver’s occupational/schooling status clarified; Added categories for Govt, Private, Self-employed, Student for both care-giver and patient
Section 6: Household expenditure	Add rows for miscellaneous costs and mobile/internet expenses	“Miscellaneous” and “Mobile/Internet” cost rows included

**Table 2:** Section-wise item distribution, Content Validity Ratio (CVR) and Content Validity Index (CVI) (Rounds 2 and 3)

Section	Items (Round 2)	Items (Round 3)	CVR range (Round 2)	CVR range (Round 3)	Overall CVI (Round 2)	Overall CVI (Round 3)	Notes
1. Sociodemographic	14	14	–	–	–	–	Not included in validity/reliability testing; factual items only
2. Treatment seeking behaviour	8	6	0.65–0.92	0.82–0.95	0.78	0.92	Some items below 0.75 CVR removed in Round 3
3. Outpatient costs	18	15	0.60–0.89	0.80–0.92	0.77	0.89	Items with low CVR removed; Final version: 10 items direct medical + 5 items direct non-medical
4. Inpatient costs	20	16	0.62–0.87	0.79–0.90	0.76	0.88	Items with low CVR removed; Final version-2 items screening + 9 items direct medical + 5 items direct non-medical
5. Indirect costs	9	6	0.60–0.85	0.81–0.90	0.75	0.86	Items with low CVR removed or reworded; Includes productivity loss and caregiver costs
6. Household expenditure & financing	18	17	0.68–0.92	0.83–0.96	0.79	0.93	Items with low CVR removed; Data used for catastrophic health expenditure estimation
Total CHEQ (Sections 1–6)	87	74	–	–	0.78	0.92	Total reflects removal of 14 low-CVR items and one additional item added for consolidated cost
7. Sequelae (HEAL-AFI only)	2	2	0.82–0.90	0.82–0.90	0.82	0.90	Follow-up module for sequelae; OPD/IPD/indirect costs repeated as 7a–7c
Total HEAL-AFI (Sections 1–7)	89	76	–	–	0.79	0.92	Final HEAL-AFI total includes sequelae module

Round 3: re-assessment of content validity and translation

The 73-item draft was re-evaluated in Round 3. Experts recommended avoiding double-counting across cost domains, adding a consolidated direct medical cost item for participants unable to itemize each component (e.g., bundled costs at private clinics), and also rewording indirect cost items to enhance cultural and contextual relevance. These changes increased the final item count to 74 in the CHEQ tool and 76 in HEAL-AFI, which includes a sequelae-specific follow-up section. All items met the CVR threshold in Round 3, and the overall CVI improved to 0.92, indicating excellent content validity. Table 2 summarizes the section-wise progression of CVR and CVI values across both rounds.

Round 4: face validity and feasibility

Round 4 evaluated face validity and feasibility with 32 community participants and eight field interviewers. The participants rated clarity, ease of understanding, difficulty, and time burden using a four-point Likert scale. Most found the tool clear and easy to understand, although minor challenges were noted in recalling indirect costs, particularly caregiver time and productivity losses. Interviewers echoed these concerns but reported that administration was generally smooth, with logical flow and appropriate skip patterns. A few items in the non-medical and indirect cost domains required clarification or probing but did not affect overall usability. The mean administration time was 24.3 ± 5.1 minutes during the first test (T1) and 23.7 ± 4.8 minutes at retest after 12 days (T2), indicating a stable administration time. Table 3 presents detailed Likert-scale responses from both participants and interviewers.

**Table 3:** Face validity and feasibility assessment (32 participants and eight interviewers)

Domain	Response option	Participants (%)	Interviewers (%)
Clarity of items	Very easy	43.8	50.0
	Easy	46.8	50.0
	Difficult	6.3	0
	Very difficult	3.1	0
Ease of understanding and observed participant ease of understanding	Very easy	37.5	62.5
	Easy	50.0	37.5
	Difficult	9.4	0
	Very difficult	3.1	0
Ease of administration (interviewer-rated)	Very easy	-	75.0
	Easy	-	25.0
	Difficult	-	0
	Very difficult	-	0
T1: Test Mean time (minutes ± SD)	24.3 ± 5.1		
T2: Retest mean time (minutes ± SD)	23.7 ± 4.8		

Round 5: reliability

Test-retest reliability was assessed after a 12-day interval among the same 32 participants. Categorical variables such as type of first provider and source of health financing demonstrated substantial reproducibility, with Cohen’s κ ranging from 0.63 to 0.79. Observed agreement was consistently high (72-84%), while the expected agreement ranged from 19% to 27%, supporting the robustness of categorical measures. Continuous variables, including direct medical expenditures, travel costs, and travel time, demonstrated excellent reproducibility with ICCs between 0.82 and 0.94. This indicates minimal variation between test and retest measures and high participant consistency in reporting cost-related details. Internal consistency was strong across multi-item sections. Cronbach’s α ranged from 0.78 in Section 3 (outpatient costs) to 0.89 in Section 6 (household expenditure and financing), confirming satisfactory homogeneity of items within sections. Table 4 presents detailed reliability statistics with interpretations.

**Table 4:** Reliability statistics (n = 32, test–retest interval = 12 days)

Section	Variable type	Cohen’s κ (95% CI)	Observed agreement (%)	Expected agreement (%)	ICC (95% CI)	Cronbach’s α	Interpretation
Section 2 (Care-seeking pathway)	Categorical	0.63 (0.51–0.74)	72	25	–	–	Substantial agreement
Section 3 (Direct outpatient costs)	Continuous	–	–	–	0.84 (0.77–0.89)	0.78	Good reliability; acceptable internal consistency
Section 4 (Direct inpatient costs)	Continuous	–	–	–	0.91 (0.87–0.95)	0.85	Excellent reliability; strong internal consistency
Section 5 (Indirect costs)	Mixed (categorical + continuous)	0.71 (0.60–0.80)	81	21	0.82 (0.73–0.88)	0.80	Substantial agreement; good consistency
Section 6 (Household expenditure and financing)	Continuous	–	–	–	0.94 (0.90–0.96)	0.89	Excellent reproducibility; high internal consistency
Overall tool	Mixed	0.63–0.79	72–84	19–27	0.82–0.94	0.78–0.89	Substantial to excellent reliability

## Discussion

This study reports the systematic development and validation of two complementary instruments: the generic CHEQ and the disease-specific HEAL-AFI. Together, these tools aim to capture the household-level economic burden of illness. By following a five-stage process-combining quantitative psychometric methods (CVR, CVI, Cohen’s κ, ICC, and Cronbach’s α) with iterative qualitative feedback from experts and community participants-the instruments demonstrated high content validity, internal consistency, and reproducibility.

The application of the Lawshe method for CVR estimation provided a rigorous foundation for content validation [20]. A panel of eight experts, consistent with the recommended range of five to ten experts for meaningful consensus building [25], evaluated the items in multiple rounds. Notably, several items failed to meet the CVR threshold in Round 2 but showed substantial improvement after item refinement, with the final CVI reaching 0.92. This iterative approach underscores the importance of structured expert feedback in enhancing item relevance and clarity.

Reliability testing further established the tools’ robustness. Cohen’s κ values for categorical items ranged between 0.63 and 0.79, with observed agreement exceeding 70%, indicating substantial reliability. ICC values between 0.82 and 0.94 demonstrated excellent reproducibility for continuous variables, and Cronbach’s α values between 0.78 and 0.89 confirmed high internal consistency. These metrics affirm that both CHEQ and HEAL-AFI reliably capture cost data across diverse domains and item types.

By contrast, earlier validated questionnaires such as CoPaQ [15] demonstrated limited standardization in cost domains, ambiguous recall periods, and the absence of structured reliability testing. CHEQ introduces several methodological improvements, including clearly defined cost domains, standardized recall periods, and a structured psychometric framework. Additionally, it is designed for use within prospective data collection settings to improve the accuracy of cost estimation. Longo et al.’s instrument for cancer patients excluded productivity loss due to privacy concerns and omitted data on household financial liabilities such as debt and mortgage payments [17]; CHEQ includes both these elements. Schweikert et al.’s cost tool for cardiac patients adopted a societal perspective [19], while CHEQ emphasizes the household and patient perspectives - crucial for understanding out-of-pocket expenditures in low-income settings. Similarly, the WHO TB cost tool was limited to DOTS-enrolled patients [26], potentially excluding those deterred by high costs from seeking care. In contrast, CHEQ and HEAL-AFI were designed for community-level administration and captured data even from households using informal or traditional care pathways-a common scenario in underserved urban settlements [14].

HEAL-AFI extends CHEQ’s utility by incorporating geospatial data and modules for sequelae-related costs - elements rarely included in existing tools. While intangible costs such as emotional distress, as captured by Fox et al. [18], were beyond the scope of these instruments, the inclusion of sequelae-related expenses in HEAL-AFI marks a meaningful step toward comprehensiveness. Together, CHEQ and HEAL-AFI offer a more complete and context-sensitive method for capturing the economic burden of illness at the household level.

Strengths

The primary strength of this study lies in its methodologically rigorous and contextually sensitive design that can be administered in a community setting. The five-stage validation framework ensured a high level of content validity, drawing on both expert consensus and real-world community input. CHEQ systematically captures a full range of household costs, including direct medical, direct non-medical, indirect, and sequelae-related expenses, providing a holistic view of illness burden. The tools also incorporate colloquial terminology and electronic skip patterns, enhancing respondent comprehension and reducing burden. Real-time data capture reduced recall bias, while average completion times of under 25 minutes ensured operational feasibility in field settings.

The adaptability of CHEQ was demonstrated through its successful transformation into the disease-specific HEAL-AFI, which integrates geospatial mapping to explore cost implications alongside healthcare-seeking behaviour. This flexibility enhances the tool’s relevance for both chronic and acute conditions and across diverse population settings. Importantly, when applied within prospective data collection settings, the instrument allows for improved estimation of economic burden compared to retrospective approaches. In addition, repeat testing supported the instrument’s reliability over time.

Limitations

Several limitations must be acknowledged. First, validation was conducted at a single urban resettlement site in Delhi, potentially limiting generalizability to rural, tribal, or more affluent urban populations. The sample size used for face and test-retest validity (n = 32), although sufficient for preliminary psychometric evaluation, reduces the precision of statistical estimates and limits subgroup analysis. While hospital records were used to cross-verify inpatient expenditures when available, broader external validation with billing data or longitudinal household expenditure tracking was not undertaken. Additionally, although the instrument is intended for use in prospective data collection to minimize recall bias, the study did not assess the extent of recall bias that may still persist. The tools do not capture intangible costs such as emotional or psychological distress, which can be substantial in chronic illness contexts. The tools were interviewer-administered, limiting self-report use. Finally, despite their modular design, the tools’ performance in non-urban or culturally different settings remains to be empirically demonstrated.

Scope and implications

The CHEQ and HEAL-AFI instruments address a major gap in the standardization and reliability of tools for measuring household economic burden of illness in India and similar settings. CHEQ’s modular structure allows context-specific adaptation, making it suitable across diseases and diverse populations. In addition to capturing direct and indirect costs, CHEQ includes treatment-seeking behavior, enabling analysis of how financial constraints shape care pathways. HEAL-AFI, developed as a disease-specific adaptation of CHEQ, was validated alongside the generic tool in this study. It demonstrates contextual flexibility through its focus on AFIs such as dengue and chikungunya, and introduces geospatial data to link economic burden with healthcare access and transmission dynamics. While this paper reports the development and validation of both instruments, findings from the main HEAL-AFI cohort study will be presented separately. Together, CHEQ and HEAL-AFI address a significant methodological gap in the standardization and reliability of tools for measuring the household economic burden of illness in India and similar settings. They support robust data collection on out-of-pocket spending, financial coping strategies, and vulnerability to catastrophic health expenditure. Wider implementation in rural and multi-disease contexts, expanded validation cohorts, along with linkage to facility billing data, may strengthen external validity and policy relevance.

## Conclusions

This study presents the systematic development and validation of two complementary instruments-CHEQ and HEAL-AFI-for capturing household-level health expenditures. Both tools demonstrated strong content validity, high reliability across cost domains, and operational feasibility in low-income urban settings. By combining structured cost categories with contextual adaptations such as colloquial phrasing, sequelae modules, skip patterns, and geospatial mapping, they offer a comprehensive and flexible framework for estimating the economic burden of illness. Their application holds significant potential to inform equitable health financing policies, strengthen financial risk protection, and support evidence-based planning in India and comparable resource-constrained settings.

## Appendices

**Table 5:** Study tool

Section 1: Sociodemographic profile of the patient									
S. No.		Parameter				Options			
1.		Name							
2.		Age (in years)							
3.		Gender				1. Male			
						2. Female			
						3. Others			
4.		Address							
5.		Religion:				1. Hindu			
						2. Muslim			
						3. Others (specify)			
7.		Education of the head of household				1. Illiterate/can only read but not write			
						2. Primary school (nursery/KG, Anganwadi, Class 1st-7th/can read and write)			
						3. Middle school (Class 8th-9th)			
						4. High school (Class 10th-11th)			
						5. Intermediate or diploma (Class 12th pass, 12th pass with diploma)			
						6. Graduate (except technical graduation degrees): BA, B.Sc., B.Ed			
						7. Profession or honors (Ph.D)/post-graduate degrees (any master’s degree)/ technical graduation degrees (MBBS, B.Arch, B.Tech)			
8.		Occupation of the head of household				1. Unemployed/students/retired without any pension			
						2. Unskilled worker (no training required): domestic servants/peon/watchman			
						3. Semi-skilled worker (some training required): factory labourer/car-cleaner/ small shopkeeper/ machine operators/assemblers			
						4. Skilled worker (training required): driver/ telephone operator/ carpenter/ mechanic/ mistri/ painter/ craft and related trade workers			
						5. Arithmetic skills job: clerk/small accountant/typist/primary school teacher/farm owner/shopkeeper/salesman/insurance agent/skilled agriculture and fishery workers/field workers – news journalist, research purpose, etc.			
						6. Semi-professional (requires high school education but a routine job) - high school teacher/junior administrator/small business owner			
						7. Technicians/associate professionals/medium business owners			
						8. Professional – doctors/engineers/architects/ directors/managers/senior administrators/college principal/bank managers/senior officers/chartered accountant (CA)/large business owners			
9.		House type				1. Kutcha			
						2. Pucca			
10.		Ownership of the house				1. Own house			
						2. On rent			
11.		Social caste				a) SC			
						b) ST			
						c) OBC			
						d) UR/general			
						e) Not willing to report			
12.		Migration status				1. Migrated within the last 10 years			
						2. Migrated and staying for more than 10 years			
						3. Not applicable/not migrated			
13.		Possess a ration card				1) Yes			
						2) No			
		If yes, what is the colour of the ration card?							
14.		Total monthly family income (of all the earning members)							
15.		Number of family members							
Section 2: Treatment-seeking behaviour for acute fever									
1.		What was the date of onset of fever?							
2.		What was the FIRST action that you took when you were experiencing this fever episode?				1. Consulted a physician			
						2. Consulted a traditional practitioner (Bengali doctor/quack, spiritual healer, etc.)			
						3. Consulted a pharmacist at the pharmacy outlet			
						4. Self-medication (home remedies, self-diagnosis, and search on the Internet, using previously prescribed medication, etc.)			
						5. Did nothing			
						6. Others (specify)			
If the response to Q2 is (1) or (2), proceed to Q3. If the response to Q2 is (3) or (4), skip to Q4 and Q5. If the response to Q2 is (5) (6), skip to Q5.									
3.		If the response to Q2 is (1) or (2), give the following details of the first visit, as well as any subsequent visits.							
3a.		Day of fever (Day 1, Day 2, Day 8, etc.)							
3b.		System of healthcare				1. Allopathic			
						2. AYUSH (Ayurvedic/ Homeopathic/Siddha/Unani)			
						3. Others (Quack/spiritual healers/Bengali doctor, etc.)			
3c.		Type				1. Government			
						2. Private			
						3. Others			
3d.		Time taken to reach the place of treatment				1. Less than 15 min			
						2. More than 15 min-30 min			
						3. More than 30 min-1 hour			
						4. >1 hour			
3e.		Distance to reach the place of treatment				1. Less than 5 km			
						2. 5-10 km			
						3. 11-15 km			
						4. >15 km			
4.		How many times have you self-medicated for a fever episode in the past week?				1. Once			
						2. Twice			
						3. Thrice			
						4. More than three times			
						5. None			
5.		What were the reasons? (Multiple answers possible)				Ailment not considered serious			
						Financial problems			
						Lack of trust over medicine efficacy			
						Lack of trust over healthcare providers			
						Travel distance is more			
						Waiting time is more			
						Have to take leave from official work			
						No replacement person for household works			
						Nobody to accompany to the healthcare facility			
						Others (specify)			
						Ailment not considered serious			
6.		Whether covered by any scheme for health expenditure support for a fever episode?				Government/PSU as an employer (e.g. CGHS, reimbursement from govt. etc.)			
						Employer-supported (other than govt./PSU) health protection (e.g. ESIS)			
						Private insurance companies			
						Not covered			
						Others (Specify)			
6a		If the answer to Q6 is 1) or 2), specify:							
6b		What was the amount reimbursed during this fever episode? (Specify)							
Section 3: Outpatient department cost									
Item/particular			Unit cost (Rs)				No. of times/no. of units	Any remarks (e.g., source (Pvt/Govt/NGO)	
3A. Direct medical cost:									
Hospital fees (registration fee/doctor/nurse fee)									
Telemedicine (any online consultation)									
Medicines (allopathic)									
Medicines (AYUSH, etc.)									
Consumables (e.g. gloves, IV fluid apparatus, injections, etc.)									
Equipment purchased during this period (e.g. BP machine, thermometer, etc.)									
Investigation costs (pathological/microbiological/biochemical/biochemical investigations)									
Investigation costs (radiological)									
Miscellaneous (e.g. any emergency/casualty costs)									
Consolidated amount (if breakup not available)									
Mention details of the particulars of the consolidated amount									
3B. Direct non-medical cost:									
Transportation									
Traditional practitioner (Bengali doctor/quack, spiritual healer, etc.)									
Special diet during this period (e.g. coconut water, giloy, fruits, etc.)									
Support services (payment given to the health assistant/attendant in the hospital/OPD)									
Miscellaneous (religious faith, e.g., any cost during worship for a cure from illness)									
Section 4: Inpatient department cost (record-basis)									
1.		Have you been admitted for this fever episode in an hospital?	1. Yes						
			2. No						
2a.		Date of admission							
2b.		Date of discharge							
Item/ particular			Unit cost (Rs)			No. of times/No. of units		Any remarks (e.g., source (Pvt/Govt/NGO)	
4A. Direct medical cost:									
Hospital fees (registration fee/doctor/nurse/hospital attendant fee)									
Medicines									
Consumables									
Equipment purchased during this period									
Investigation costs (pathological/microbiological/biochemical investigations)									
Investigation costs (radiological)									
Blood/platelet transfusion									
Physiotherapy									
Miscellaneous (any emergency/casualty costs, room charges, ICU cost, etc.)									
4B. Direct non-medical cost:									
Transportation (ambulance charges, own vehicle charges, public transport, etc.)									
Diet (inclusive of both the patient and caregiver for the duration of the hospital stay)									
Support services (caregiver cost/assistant cost/ASHA)									
Lodging expenses									
Miscellaneous (religious faith, e.g. any cost during worship for a cure from illness)									
Section 5: indirect cost									
1a.		Is the patient employed?			1. Yes				
					2. No				
1b.		If the answer to Q.1a. is ‘Yes’, specify the sector of employment.			1. Government sector				
					2. Private sector				
					3. Self-employed (business)				
2.		If the answer to Q.1a. is ‘Yes’, what is the patient’s earnings for one day? (INR)							
3.		If the patient is school-going, how many school days are missed?							
4a.		Ability to perform usual activities during the course of illness (excluding children) (day-wise): 1 = completely unable; 2 = partially unable; 3 = completely able			Day-1:				
					Day-2:				
					Day-3:				
					Day-4:				
					Day-5:				
4b.		If partially unable, mention the number of hours in a day that the person was not able to perform usual activities.			Day-1:				
					Day-2:				
					Day-3:				
					Day-4:				
					Day-5:				
5.		If the patient was unable/partially unable to perform usual activities due to illness, who assisted during the course of illness?							
		Caregiver-1			Relation to patient				
					Occupation (1 = employed, 2 = unemployed)				
					If employed, what is their monthly salary? (INR)				
					Did they have to miss workday/ cut-off on the usual activities? [1 = Yes, missed complete work-day; 2 = Yes, had to cut-off on usual activities; 3 = No]				
		Caregiver-2			Relation to the patient				
					Occupation (1 = employed, 2 = unemployed)				
					If employed, what is their monthly salary				
					Did they have to miss workday/ cut-off on the usual activities? [1 = Yes, missed complete work-day; 2 = Yes, had to cut-off on usual activities; 3 = No]				
6a.		Do you have a paid house-help/maid?			1. Yes				
					2. No				
6b.		During the course of illness, did you hire any additional help or pay extra to the already hired house-help?			1. Yes				
					2. No				
6c.		If yes, provide details of the helper.							
		Helper-1			Type of helper				
					Monthly salary:				
					No. of extra hours in a day assisted apart from usual work				
					Any other form of compensation given? (e.g., lump sum amount of money; food items, etc.) [1- Yes, 2- No]				
					If yes, details (in monetary terms)				
		Helper-2			Type of helper				
					Monthly salary:				
					No. of extra hours in a day assisted apart from usual work				
					Any other form of compensation given? (e.g. lump sum amount of money; food items, etc.) [1- Yes, 2- No]				
					If yes, details (in monetary terms)				
Section 6a: Average expenditure per month (irrespective of fever episode)									
Item/particular				Cost (in Rs.)					
House rent									
Electricity bills									
Water bills									
Mobile and/or internet bills									
Medical expenses									
Insurance									
Health insurance									
Food									
Clothing									
Education									
Transport/fuel									
Substance abuse: tobacco									
Substance abuse: alcohol									
Miscellaneous expenditure (repair work, maintenance, etc.)									
Others									
Section 6b: Source of finances for overall expenses									
1.	Major source of finance for expenses			Household income/ savings					
				Borrowings					
				Sale of physical assets					
				Contributions from friends and relatives					
				Other sources					
2.	Second most important source of finance for expenses			Household income/ savings					
				Borrowings					
				Sale of physical assets					
				Contributions from friends and relatives					
				Other sources					
3.	Are you covered by any scheme for overall health expenditure support?			Government/PSU as an employer (e.g. CGHS, reimbursement from govt. etc.)					
				Employer-supported (other than govt./PSU) health protection (e.g. ESIS)					
				Private insurance companies					
				Not covered					
				Others (specify)					
Section 7 is applicable only for patients with a laboratory-confirmed diagnosis of dengue and chikungunya.									
Section 7: sequelae/complications									
Follow-up done telephonically weekly for the first month and bi-weekly for the second month									
1.	Are you experiencing any of the following symptoms post-recovery from dengue/chikungunya? (Multiple options possible)			Fatigue					
				Somnolence (excess sleeping)					
				Headache					
				Joint pain					
				Rash					
				Concentration impairment					
				Memory impairment					
				Nausea/vomiting					
				Hair loss					
				Others (specify)					
2.	Are you taking any treatment (e.g. physiotherapy for joint pain) for these symptoms?			1. Yes					
				2. No					
If yes, proceed to further sections 7a, 7b, and 7c.									
Follow-up module for sequelae; OPD/IPD/indirect costs repeated as Section 7a–7c.									
